# Relationship between the abnormal diastolic vortex structure and impaired left ventricle filling in patients with hyperthyroidism

**DOI:** 10.1097/MD.0000000000006711

**Published:** 2017-04-28

**Authors:** Bin-Yu Zhou, Ming-Xing Xie, Jing Wang, Xin-Fang Wang, Qing Lv, Man-Wei Liu, Shuang-Shuang Kong, Ping-Yu Zhang, Jin-Feng Liu

**Affiliations:** a Department of Ultrasound, Union Hospital, Tongji Medical College, Huazhong University of Science and Technology, Wuhan; b Department of Ultrasound, Shandong Provincial Qianfoshan Hospital, Shandong University, Jinan, China.

**Keywords:** diastolic function, echocardiography, filling, hyperthyroidism, left ventricular, vector flow mapping

## Abstract

Intraventricular hydrodynamics plays an important role in evaluating cardiac function. Relationship between diastolic vortex and left ventricular (LV) filling is still rarely elucidated. The aim of this study was to evaluate the evolution of vortex during diastole in hyperthyroidism (HT) and explore the alteration of hydromechanics characteristics with sensitive indexes.

Forty-three patients diagnosed with HT were classified into 2 groups according to whether myocardial damage existed: simple hyperthyroid group (HT1, n = 21) and thyrotoxic cardiomyopathy (HT2, n = 22). Twenty-seven age- and gender-matched healthy volunteers were enrolled as the control group. Offline vector flow mapping (VFM model) was used to analyze the LV diastolic blood flow patterns and fluid dynamics. Hemodynamic parameters, vortex area (A), circulation (C), and intraventricular pressure gradient (Δ*P*), in different diastolic phases (early, mid, and late) were calculated and analyzed.

HT2, with a lower E/A ratio and left ventricular ejection fraction (LVEF), had a larger left atrium diameter (LAD) compared with those of the control group and HT1 (*P* < .05). Compared with the control group, the vortex size and strength, intraventricular pressure gradient during early and mid-diastole were higher in HT1 and lower in HT2 (*P* < .05). And in late diastole, the vortex size and strength, intraventricular pressure gradient of HT2 became higher than those of the control group (*P* < .05). Good correlation could be found between C_E_ and E/A (*P* < .05), C_M_ and ΔPM (*P* < .01), C_L_ and FT3 (*P* < .05).

VFM is proven practical for detecting the relationship between the changes of left ventricular diastolic vortex and the abnormal left ventricular filling.

## 1 Introduction

Previous studies on left ventricle (LV) filling dynamics focused on single-point flow velocity measurements by Doppler echocardiography. However, the single-point velocity measurements cannot accurately evaluate the global diastolic function of LV because the intraventricular velocity profile is complex.^[1]^ LV diastolic vortex has been suggested to be essential for efficient pumping function, with a better understanding to the intraventricular hydrodynamics, while altered vortex was associated with LV pathologies.^[2]^ Intraventricular vortex formed by the morphology and function of the cardiovascular system can reveal the global diastolic function of LV and the exceptional adaptability of cardiovascular system for maintaining the blood circulation relatively constant under a wide range of workload.^[3]^

Computational fluid dynamics (CFD) and particle image velocimetry (PIV) have been applied in some new technologies for the assessment of cardiac function that have expanded our options to visualize and analyze the complex intracardiac flow. Among them, cardiac magnetic resonance (CMR), CE-PIV technique combined contrast echocardiography (CE) and PIV, vector flow mapping (VFM) based on color Doppler flow imaging (CDFI) and speckle tracking are the principal methods that are currently used in the assessment of patients in the very early stage of cardiovascular disease or for investigating complex geometry after cardiac surgery.^[4,5]^ CMR displays 3-dimensional (3D) chamber flow fields in a variety of visualization methods via electrocardiogram (ECG) and respiratory gating, but it has not yet been routinely used in clinical practice due to long duration of the test, a rather high cost, and a low temporal resolution.^[6]^ Compared with CMR, CE-PIV has superior temporal resolution. It uses moving tracks of ultrasound microbubbles to represent the distribution of the blood flow field, thereby providing a more accurate calculation of the flow velocity. However, the test requires contrast media, and the range of velocities that can be measured is limited.^[7]^ VFM visualizes cardiovascular blood flow in vectors based on color Doppler superimposed on speckle tracking—a method for tracking cardiac wall motion. Improvements have been made in new VFM to allow for more accurate and stable calculation of hemodynamic parameters and flow velocity near the wall. It is considered to be one of the representative methods for visualization of blood flow in vivo that can compete with CMR and CE-PIV.^[8,9]^

Excessive secretion of thyroid hormones in patients with hyperthyroidism (HT) produces cardiotoxicity.^[10]^ The evolution of the LV diastolic vortex in HT at different stages might change with the volume load, cardiac structure, and function. This study attempted to detect the LV hemodynamics in HT using VFM and investigate the relationship between the diastolic vortex field and abnormal LV filling.

## 2 Methods

### 2.1 Subjects

Sixty patients diagnosed with HT between March and December 2014 in Union Hospital, Tongji Medical College, Huazhong University of Science and Technology, China were randomly recruited in this study. Nine patients were excluded due to poor image quality, and 8 cases were excluded due to cardiac arrhythmia. Forty-three patients were involved in the study finally. Of them, 22 (8 men and 14 women) who were referred to the cardiologist and identified with dilated cardiomyopathy were assigned to thyrotoxic cardiomyopathy group (HT2), aged between 21 and 63 years (mean 42.00 ± 13.01 years). The remaining 21 patients (9 men and 12 women) were assigned to simple HT group (HT1), aged between 18 and 56 years (mean 35.45 ± 10.34 years). In addition, 27 gender- and age-matched healthy volunteers (15 men and 12 women) during the same period in our hospital were enrolled as the control group, aged between 20 and 56 years (mean 40.62 ± 13.08 years).

Diagnostic criteria for HT^[11]^ presence of classical clinical symptoms and physical signs of HT; laboratory criteria: thyroid stimulating hormone (TSH) < 0.5 μU/mL (normal range = 0.5–10 μlU/mL), free Triiodothyronine (FT3) > 9.15 pmol/L (normal range = 3.19–9.15 pmol/L), and/or free Thyroxine (FT4) > 25.47 pmol/L (normal range = 9.11–25.47 pmol/L); exception of other organic heart diseases and symptom of thyrotoxic cardiomyopathy. Thyrocardiac disease is defined as the presence of congestive heart failure, angina pectoris, or both, in a patient with hyperthyroidism.^[12]^ The study was approved by the ethics committee of Tongji Medical College, Huazhong University of Science and Technology. All study subjects provided written informed consent.

### 2.2 Image acquisition and analysis

Transthoracic echocardiography was performed on all subjects using Aloka Prosound F75 scanner equipped with a frequency 2 to 4 MHz transducer (UST5415). ECG was simultaneously recorded to determine the cardiac phase. Left atrial diameter (LAD), left ventricular end-diastolic diameter (LVEDD), transmitral peak E and A velocities (E peak, A peak) were measured at the parasternal LV long-axis view. LV ejection fraction (LVEF) was calculated by the modified biplane Simpson method. Standard 2D color flow Doppler dynamic images in 3 consecutive beats were recorded from the apical long-axis view. The scanning width, imaging depth, and spatial temporal settings were optimized to achieve the highest possible frame rate. Doppler velocity scale varied from 50 to 60 cm/s in this study, and the frame rate was set at 25 frames per second for all subjects. All recordings were stored digitally and analyzed offline.

All the stored raw data were processed on offline VFM workstation (DAS-RSI, Aloka, Japan). As the boundary of flow in LV was outlined, the system automatically traced the flow signals and calculated the velocity vector. Diastole was determined based on synchronous ECG (from the end of the T-wave to the onset of the Q-wave). Early diastole (rapid filling), mid diastole (slow filling), and late diastole (atrial systole) were defined depending on the flow rate-time graph of mitral valve (MV) and behavior of valve.^[13]^

A sampling line from the endocardium of cardiac apex to MV was drawn under the guidance of streamline to measure the intraventricular velocity gradient (Δ*V*) (Δ*V* = velocity of MV − velocity of cardiac apex). Then intraventricular pressure gradients in different filling phases (ΔP_E_, ΔP_M_, and ΔP_L_) can be calculated according to the formula Δ*P* = 4 × Δ*V*^2^.^[14]^

**Figure 1:**
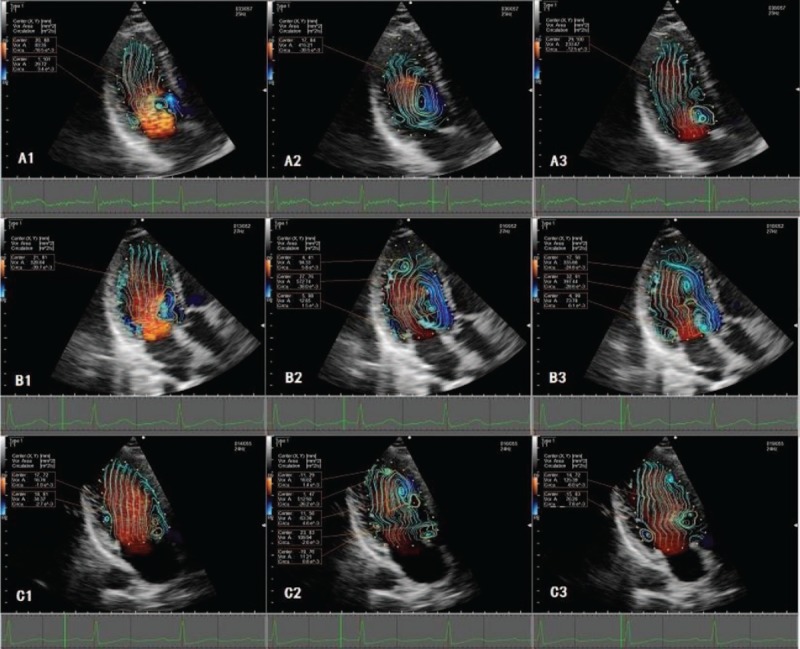
Formation and evolution of the intraventricular vortex during diastole. Vortex features displayed a typical biphasic temporal course during diastole in all 3 groups. Two vortexes appeared behind the MV leaflets during early diastole (A1, B1, and C1), and the posterior vortex in HT2 (C1) failed to be detected in an unstable condition. In mid-diastole, a relatively apically located large vortex existed in the control group and HT1 (A2, B2). Instead, several small and scattered vortexes appeared in HT2 (C2). Like early diastole, 2 symmetric vortexes appeared below MV in late diastole (A3, B3, and C3).

2D streamline map and vortex map (Fig. 1) were acquired after processing, so the evolution of the diastolic LV vortex can be intuitively and quantitatively illustrated. The vortex area (A), and total vorticity, namely circulation (C) were automatically tracked and displayed by the analysis software. C is equivalent to the integral of the normal component of “vorticity” (ω) on an arbitrary plane (S) enclosed by a closed curve using the following formula. 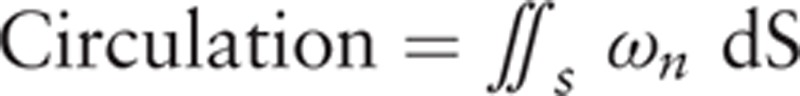


It is calculated by accumulating the velocity component of a tangent on a closed curve, thus C becomes higher when the flow velocity is higher. Dynamic evolution of A and C was observed in different phases. Since the 2 vortices below the MV were not stable enough in both early and late diastole (especially the vortex below the posterior leaflet), only the one near the anterior leaflet was analyzed.^[13]^

Observer repeated the same measurements in randomly selected 12 cases after 1 week, and another independent observer repeatedly performed measurements on these 12 subjects. Then, intra- and interobserver reproducibility of Δ*P* and C for both filling phases were analyzed.

### 2.3 Statistical analysis

SPSS Statistic software (version 20.0 IBM SPSS, Chicago, IL) was used to perform statistical analysis in the study. Continuous data were expressed as mean ± SD (x ± s). Clinical characteristics and hemodynamic variables were compared using one-way analysis of variance (ANOVA) followed by Dunnett contrasts against the control group, when appropriate. Relationships between vortex variables and clinical, echocardiographic characteristics were evaluated by Pearson linear regression analysis. Intra- and interobserver variability values were analyzed by Bland–Altman bias plots. The mean and intraobserver and interobserver differences were plotted against the mean of 2 measurements, with 95% of the data points recommended to lie within ± 2SDs of the mean difference. *P* < .05 was considered statistically significant.

## 3 Results

### 3.1 Clinical and echocardiographic characteristics

The clinical data and hemodynamic variables are described in Table 1. There were no significant differences in age, body surface area (BSA), and systolic blood pressure (SBP) among these 3 groups (*P* > .05). HR, SBP, FT3, and FT4 were higher, and TSH was lower in patients compared with those of the controls (*P* < .05). Patients in HT1 had the shorter course and faster HR than those in HT2. Compared with controls and HT1, HT2 showed significant increases in LAD, LVEDD, and A peak, along with E peak, E/A ratio, and LVEF reduced (*P* < .05). LVEF in HT1 was higher than that of controls, and there were no significant differences in LAD, LVEDD, E peak, A peak, and E/A ratio between them.

**Table 1:**
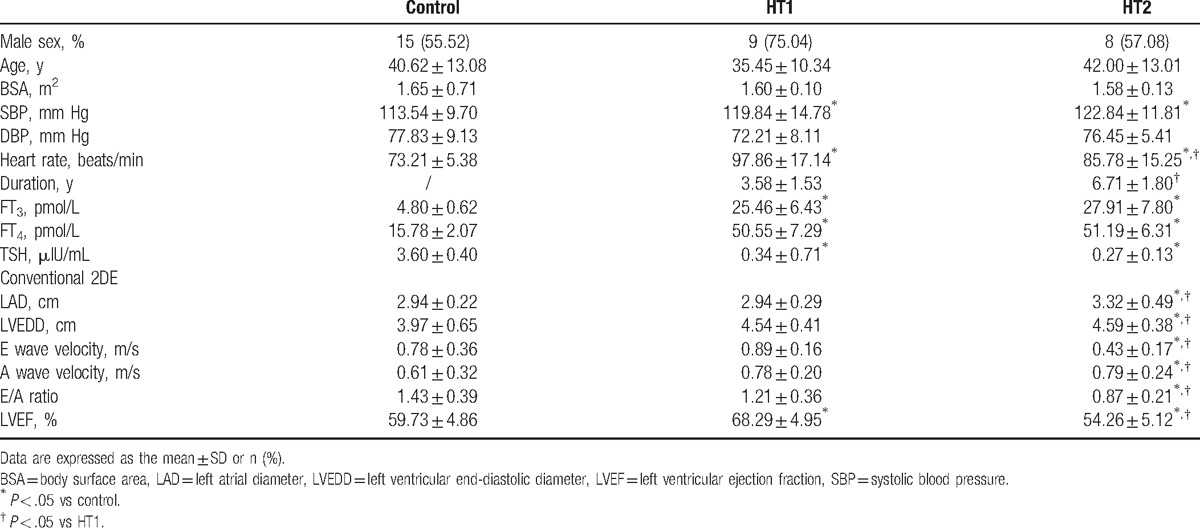
Clinical and echocardiographic characteristics between three groups.

### 3.2 Diastolic intraventricular pressure gradients

Table 2 summarizes the intraventricular pressure gradients calculated from the intraventricular velocity gradient. Compared with the control subjects, the intraventricular pressure gradients were significantly higher in HT1 during all filling phases (Δ*P*_E_, Δ*P*_M_, and Δ*P*_L_). In addition, intraventricular pressure gradients during early and mid-diastole (Δ*P*_E_ and Δ*P*_M_) in HT2 were lower than those in controls and became notably higher than controls at the end of diastole (Δ*P*_L_) (*P* < .05).

**Table 2:**
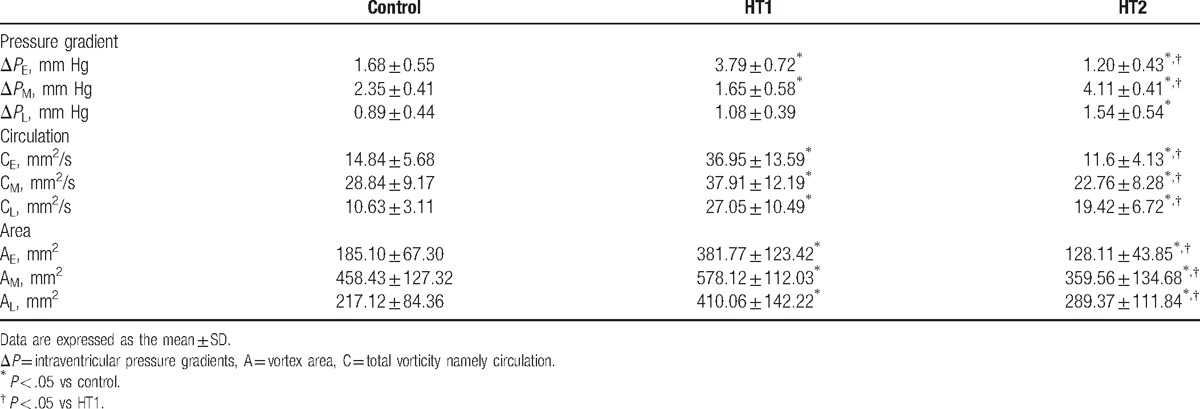
Vortex quantification parameters between three groups (x ± s).

### 3.3 Evolution of intraventricular vortices

In the control group, a strong inflow jet directed almost straight to the apex with a minor zigzag pattern and 2 asymmetric vortices appeared behind the MV leaflets after MV opening during early diastole. The one near the anterior side was larger. These 2 vortices continued their development during the period of rapid filling, reaching their full development when the E wave approached its peak (E peak). During slow filling, E wave deceleration occurred, and pressure across the MV decreased. Direction of the LV blood flow changed from apex to LV outflow, and the streamlines of inflow blood became rotated turbulently. These 2 vortices evolved into a single large one that was loosely concentrated, progressing toward the apex with the vortex area and circulation continuously growing. Followed by a late filling of atria contraction, the early filling vortex was weakened by the A wave onset, causing the late filling jet (A peak) to destroy the relatively apically located large vortex, creating another 2 symmetric and smaller vortices close to the mitral tip (Fig. 1).

**Figure 2:**
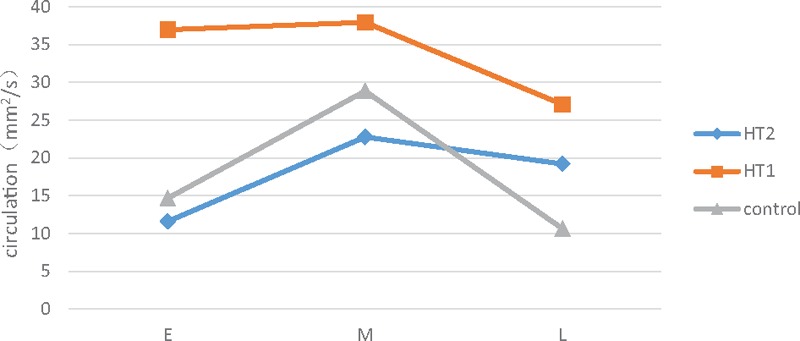
Evolvement of circulation in different diastolic phases. In early diastole, circulation of controls was weaker than those of HT1 and stronger than those of HT2. Circulation of these 3 groups increased in mid diastole concurrently. During the end of diastole, circulation in HT2 developed higher than the control group but still below HT1. The differences among the 3 groups were significant (*P* < .05).

These evolution patterns of intraventricular vortex were similar in both the control group and HT groups. The single large vortex could not be explored in some patients with simple HT due to fast HR and short duration of mid diastole. Vortical flow fields in HT1 maintained high level that the vortex propagation area and circulation were higher compared with controls and HT2 across all the phases (*P* < .01) (Fig. 2). In addition, the vorticity in HT2 evolved in a relatively unstable condition in early diastole, and the vortex below posterior leaflet failed to be detected frequently. In mid diastole, instead of a large vortex, several small and scattered vortices appeared. Vortex area and circulation in HT2 were persistently lower than control group (*P* < .05) from early to mid-diastole, but became significantly higher than controls during late filling (*P* < .01; Table 2). The 2 vortices below the MV during late diastole were more stable than those during early diastole in HT2.

### 3.4 Influencing factors of the vortex

Relationships between vortex variables and clinical, echocardiographic characteristics evaluated by Spearman linear regression analyses are shown below. C_E_ was positively correlated with E peak and E/A ratio (*r* = 0.461, 0.451; *P* < .05), and negatively correlated with DBP (*r* = −0.424; *P* < .05). C_M_ was closely correlated with Δ*P*_M_ (*r* = 0.415, 0.446; *P* < .05). C_L_ was positively correlated with FT3 and FT4 (*r* = 0.411, 0.421; *P* < .05). There were positive correlations between Δ*P*_E_ and E peak (*r* = 0.524, *P* < .05), Δ*P*_M_ and E/A ratio (*r* = 0.457, *P* < .05), Δ*P*_L_ and A peak (*r* = 0.485, *P* < .05).

### 3.5 Inter- and intraobserver variation

**Figure 3:**
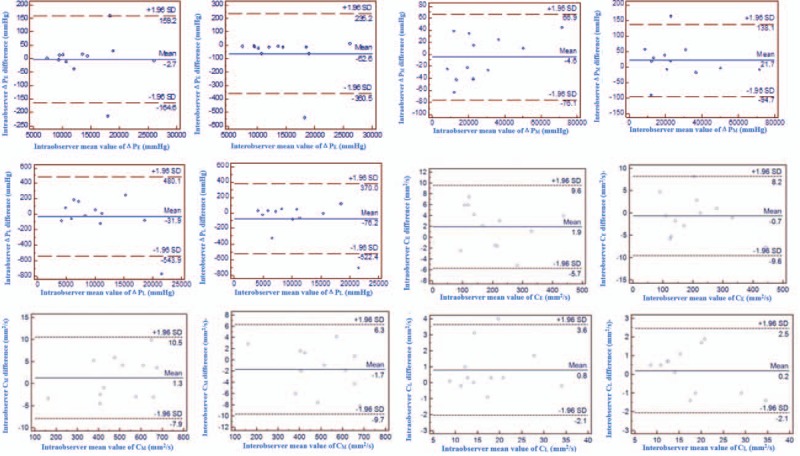
Bland–Altman plots of intraobserver (left) and interobserver (right) showing variability of diastolic Δ*P* and circulation. Average values of the measurements are plotted against the difference in the measurements. The arithmetic mean (continuous line) and 95% limits of agreement (equal to ± 1.96 SD; dotted lines) are determined.

Results of inter- and intraobserver analysis for the assessment of diastolic intraventricular pressure gradients and circulation are presented in Table 3 and Fig. 3. There were no statistically significantly differences between the 2 observers or within an observer for the quantitative vortex parameters. Interobserver analysis revealed intraclass correlation coefficients (ICCs) higher than or equal to 0.91. Intraobserver analysis showed ICCs higher than or equal to 0.86.

**Table 3:**
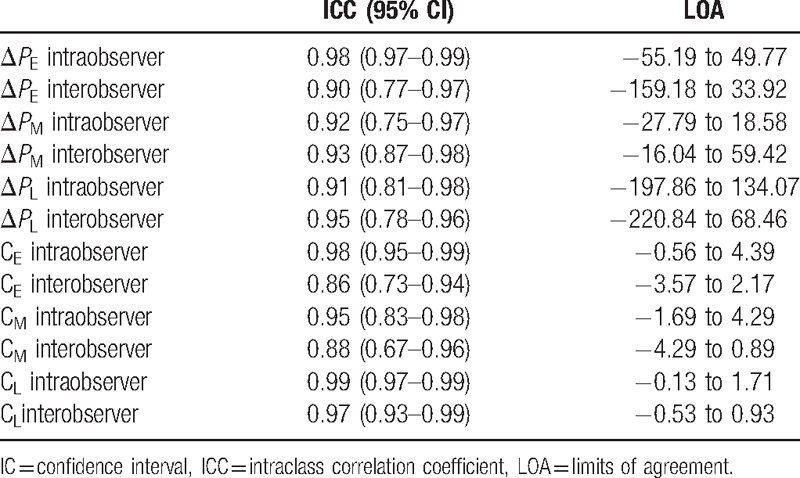
Reproducibility analysis for Δ*P* and C.

### 3.6 Discussion

HT is a common metabolic disorder with prominent cardiovascular manifestations. The direct toxic effect of thyroid hormone and indirect action via catecholamine on the cardiovascular system can lead to a typical hyper-dynamic circulatory state which manifested as accelerated HR, increased blood volume, increased stroke volume (SV), increased LVEF, and high cardiac output.^[15]^ With long-lasting excessive thyroid hormone, functional and morphological abnormalities occur in the heart, and compensatory cardiac hypertrophy exists which ultimately leads to heart failure.^[16]^ Congestive heart failure is the well-recognized complication of HT. Research showed that diastolic dysfunction appeared earlier in patients with hyperthyroid heart failure than those with hypothyroid heart failure.^[17]^ However, pathologic studies of HT generally believed that thyroid hormone enhances LV diastolic filling.^[15,18]^ Indices of conventional echocardiography are vulnerable to the geometry of the heart, HR, and load. Thus, they cannot objectively reflect the changes in diastolic function. Hydromechanics research demonstrates that intraventricular vortex reflects the LV functional status.^[19]^ Study about the relationship between LV filling and diastolic vortex in patients with HT from the perspective of fluid mechanics is rare. In this paper, we attempted to observe the characteristics of diastolic vortex in patients with HT using VFM to objectively reflect the dynamic changes in LV diastolic function.

This study showed that patients in HT1 experienced accelerated HR and increased LVEF, which were consistent with the previous studies.^[17]^ These changes were mainly due to the excess thyroid hormones that directly or indirectly affected the heart via sympathetic nerve and improved the sensitivity of myocardial β receptors to catecholamine, which resulted in cardiac sympathetic nerve hyperactivity. Therefore, the heart was in a hyperdynamic circulation state with high cardiac output, accelerated HR, and increased contraction function.^[15]^

In this study, patients with thyrotoxic cardiomyopathy had significantly longer courses than simple HT. LVEDD and LAD were higher, LVEF and E/A ratios were lower in HT2 than those in controls and HT1. These were due to the progression of the HT course. Excess thyroid hormones enhanced the calcium and glucose absorption of cardiomyocytes, accelerated metabolism, and increased the proportion of myocardial oxygen consumption. When overloading exceeded the maximum steady state, a relative myocardial ischemia and myocardial damage occurred.^[20]^ Steatosis and hypertrophy existed, LV geometric configuration and function changed, which manifested as cardiac enlargement, reduced cardiac systolic and diastolic function.

Analysis of flow field parameters showed that the vortex size and strength in these 3 diastolic phases, intraventricular pressure gradient in early and mid diastole of HT1 were higher compared with the control group and HT2, which might be associated with the improved LV filling capacity and the hyper-dynamic circulatory state. Thyroid hormones’ indirect action on the cardiovascular system accelerates HR, reduces peripheral vascular resistance, and enhances cardiac output. In addition, the direct effect of thyroid hormones on heart enhances myocardial relaxation by upregulating expression of the sarcoplasmic reticulum calcium-activated ATPase (SERCA) and downregulating expression of phospholamban.^[17,18]^ Then the larger, stronger vortex was generated in cardiac chambers. Enhanced diastolic vortex in LV can increase positive pressure and assist filling by eliminating convective deceleration load.^[21]^ And the higher intraventricular pressure gradient in patients with simple HT is helpful to pull blood from left atrium into LV more efficiently for adapting the increased circulating blood volume. Martínez-Legazpi et al^[19]^ found that diastolic vortex can assist LV filling without energy consumption and negative impact on intraventricular diastolic pressure distributions. Such vortex formation may be beneficial in efficient MV closure,^[22]^ efficient diastolic filling, minimizing kinetic energy loss,^[23]^ preventing thrombus formation,^[24]^ and maintaining normal diastolic function.^[21,25]^ The difference in E/A ratio between HT1 and controls had no statistical significance, suggesting that diastolic vortex might be more sensitive in evaluating LV filling capacity. Mintz et al^[26]^ found the enhanced left ventricle diastolic function appeared in a group of newly diagnosed and untreated HT patients, and believed that it has something to do with the early state of thyrotoxic cardiac damage. Additionally, vortices dissipate flow energy by viscous (frictional) effects, the dissipated loss increased when the vortical change speed accelerated (heart rate elevated).^[27]^ Therefore, there is a cascade of energy dissipated as heat in patients with HT, under the state of hyperdynamic circulation, which provide a favorable milieu for the development of heart failure.

The vortex size and strength, intraventricular pressure gradient in HT2 were lower compared with controls in early and mid diastole and then became higher than those of controls in late diastole. We speculated that the change of hydrodynamics might be linked to diastole dysfunction. The lasting stimulating of thyroid hormones on cardiomyocytes in patients with thyrotoxic cardiomyopathy resulted in myocardial necrosis and myocardial interstitial fibrosis, which weakened LV relaxation in early diastole. LV diastole results from the simultaneous and complementary action of relaxation and compliance respectively generated in early and late filling. The vortex generated in early diastole was too weak to ensure complete intraventricular filling. The compliance and vortex in late diastole then showed a compensatory increase during late diastole. Moreover, accelerated heart rate leads to a relative increase in the atrial contribution to left ventricular filling due to a shortened diastolic filling time. Decreased LV filling volume and increased filling pressure during early diastole may cause the syndrome of congestion in pulmonary and systemic circulation. At this time, atria systole was enhanced in patients with thyrotoxic cardiomyopathy for removing the residual blood volume in atrium and compensating the increased circulating volume, which significantly enlarged and strengthens the late diastolic vortex in LV chamber. Pasipoularide^[28]^ believed that ventricular volume load increasing could only be manifested as slight reduction in early relaxation and significant increase in late compliance, which corresponded to the results of diastolic function and vortex field in patients with thyrotoxic cardiomyopathy. In addition, the intra- and inter-observer reproducibility of VFM parameters was excellent. VFM could be used for accurate and visual evaluation of LV diastolic function and vortex field distribution in patients with HT.

Correlation analysis showed that C_E_ was positively correlated with E values and E/A ratio, indicating that the vortex in LV was related to diastolic function during early diastole. On the other hand, the abnormal vortex during active filling suggested that the diastolic dysfunction exists. C_M_ was positively correlated with Δ*P*_M,_ and Δ*P*_M_ was positively correlated with E/A ratio, showing the close correlation between vortex and intracardiac positive pressure during filling. By increasing positive pressure gradient and reducing convective deceleration load, vortices contribute to ventricular filling.^[21]^ As LV was a relatively closed chamber when MV closed during mid-diastole, ventricular pressure was not subject to left atria pressure and only associated with intraventricular vortex. C_L_ was positively correlated with FT3 and FT4, which indicated that the ventricular vortex was closely bound up with the level of thyroid hormones during left atria systole in a certain range. The excess thyroid hormones effected the relaxation of LV, which cause the left atrial pressure and vortex during late diastole rising to compensate for the rising volume load.

There were certain limitations in our study. First, manual correction for aliasing was performed in the present study, as double or higher aliasing cannot be corrected by VFM, which may underestimate the velocity. Second, color flow frame rate is slightly low. Thus, there might be deviation in the analysis of diastolic phases. Third, we failed to obtain the left atrium-ventricle pressure difference by cardiac catheterization. Therefore, the relationship between the diastolic vortex and the atrium-ventricle pressure difference could not be analyzed directly. More efforts need to be exerted in the future studies. And finally, the vortex is a 3D entity, and fluid evaluation in 3D would be desirable to ensure accurate determination of the evolution of vortex. At present, however, VFM has not been converted to 3D.

In summary, our study showed that LV flow field can give insights into abnormal diastolic filling in HT. For patients with simple HT, the improved LV diastolic function and high-performance vortex contribute to accommodating the hyper-dynamic circulatory state. In patients with obvious thyrotoxic cardiac damage, the reduced vortex during early and mid-diastole is partially related to relaxation dysfunction, and the increased vortex during late diastole is about the compensatory raising of volume load.
